# ZEB1 and Uveal Melanoma Invasiveness

**DOI:** 10.3390/ijms262110346

**Published:** 2025-10-24

**Authors:** Maria Zhilnikova, Maria Balantaeva, Sofia Zvereva, Mikhail Biryukov, Vasiliy Atamanov, Julia Poletaeva, Elena Ryabchikova, Olga Stanishevskaya, Dmitryi Chernykh, Natalia Kononova, Olga Koval

**Affiliations:** 1 Institute of Chemical Biology and Fundamental Medicine, Siberian Branch of the Russian Academy of Sciences, 630090 Novosibirsk, Russia; m.zhilnikova@alumni.nsu.ru (M.Z.); m.balantaeva@g.nsu.ru (M.B.); zvereksonik@gmail.com (S.Z.); biryukov.mm@ya.ru (M.B.); mntk@bk.ru (V.A.); fabaceae@yandex.ru (J.P.); lenryab@niboch.nsc.ru (E.R.); stanishevskaya.olya@gmail.com (O.S.); nfmntk.dima@gmail.com (D.C.); 2 Department of Natural Sciences, Novosibirsk State University, 630090 Novosibirsk, Russia; 3 S. Fyodorov Eye Microsurgery Federal State Institution, Novosibirsk Branch, 630120 Novosibirsk, Russia; 22samsonova@mail.ru

**Keywords:** uveal melanoma, personal cell cultures, ZEB1, VEGF-A, aflibercept, VEGFR2, invasiveness

## Abstract

Uveal melanoma (UM) is the most prevalent primary intraocular tumor in adults. Transcription factor ZEB1 is one of the potential master regulators of melanocytes plasticity, because it is recognized as a “driver” of epithelial-to-mesenchymal transitions (EMTs) in carcinomas. We studied the correlation of tumor invasiveness with ZEB1 status and vascular endothelial growth factor/its receptor (VEGF-A/VEGFR2) in UM cells, and also with melanocyte’s differentiation rate. Eight UM cell cultures were characterized by melanosomes content using an ETM. ZEB1, VEGF-A and VEGFR2 levels in UM cells were detected by RT-PCR, Western blot, ELISA and flow cytometry. Effects of siRNA-dependent ZEB1 knockdown on UM cell proliferation and their sensitivity to the VEGF-A inhibitor Eylea (aflibercept) were tested by MTT and in a real-time proliferation assay. UMs with an invasive growth type can maintain a high degree of melanocyte differentiation. All ZEB1^low^ cells were obtained from spindle cell tumors. The sensitivity of UM cells to Eylea inversely correlated with the level of the VEGFR2 receptor. ZEB1 knockdown completely blocked VEGF-A production while anti-VEGF treatment stimulated ZEB1 increase. In UM cell cultures, ZEB1 is a positive regulator of VEGF-A expression. In addition, there is probably a ZEB1 feedback loop that is sensitive to a drop in VEGF-A concentration. The data obtained allow us to consider ZEB1 silencing as an auxiliary link for a combined strategy of killing UM cells.

## 1. Introduction

Melanoma is a heterogeneous disease that includes tumors of various localizations. Uveal melanoma develops as a result of the malignant transformation of melanocytes of the eye uveal tract and represents about 5% of all melanomas with an average of 4.4–5.8 cases per million. It is significantly different from cutaneous melanoma, especially by its mutational burden, interaction with the immune system and type of metastases dissemination [1,2,3]. These factors complicate the study of patient-specific UM markers or prognostic factors and treatment options. Brachytherapy is the most common organ-preserving treatment of uveal melanoma, but it is limited by tumor size (large basal diameter, LBD < 9 mm) and invasive growth type [4]. Retrospective case series have demonstrated that choroidal melanomas less than 3.0 mm in LBD are highly unlikely to metastasize [5]. A metastatic form of UM has an extremely unfavorable survival prognosis [6].

Uveal melanoma disseminates hematogeneously, and the liver is the main metastatic niche, with over 90% of all incidents. Like the eye, the liver is a relatively immunotolerant organ with weak accessibility to immune cells. Therefore, innovative immunotherapeutic approaches that are effective for cutaneous melanoma, breast cancer, hematological cancers and other types of carcinomas are ineffective for the treatment of primary and metastatic forms of UM [7,8]. Therefore, alternative approaches to inducing tumor cell death are being considered, for example, “differentiation therapy” [9]. The idea is that reprogramming cancer stem cells from proliferation to differentiation could correct aberrant cell division [3]. To apply this approach, it is important to know the level of differentiation of the tumor cells. Melanocytes are specialized cells that synthesize dark pigment melanin from tyrosine in specialized lysosome-like organelles—melanosomes. Cell pigmentation reflects the number of mature melanosomes and differentiation [10]. In addition to pigmentation, the increased dendricity may also be a sign of well-differentiated melanocytes.

Based on clinical statistics, UM can be represented by cells with different phenotypes. The spindle-shaped (mesenchymal-like) phenotype of UM is less malignant than the epithelial-like one. However, no consensus exists on the phenotype-modifying factors of UM cells [11]. Since the classical morphology of melanocytes is spindle-shaped with dendritic extensions, the epithelioid phenotype implies a transition between phenotypes. For most carcinomas, an epithelial–mesenchymal transition has been described, providing tumor cells with mobility and invasiveness [12]. The master regulators of the epithelial–mesenchymal transition include the genes of the zinc-finger E-box-binding homeobox (ZEB) family. Transcription factor ZEB1 recognizes and binds to the E-boxes of epithelial gene promoter regions to decrease their expression. This directly leads to suppression of the epithelial phenotype [13]. For skin melanoma, it has recently been shown that ZEB1 controls a lineage-specific transcriptional program and phenotypic transition [14]. ZEB1 is also expressed in UM, but the role of ZEB1 in the spindle-epithelial transition of melanocytes and its contribution to invasiveness are still not completely understood. There is some data that suggests that the high expression of ZEB1 correlates with UM advancement [15]. Chen and co-authors have also recently shown that ZEB1 promotes uveal melanocyte proliferation to form large-sized tumors in vivo and spheroid in vitro [16]. Eight personal UM cell cultures were previously obtained in our laboratory from tumor samples [17,18,19,20]. Thus, based on our cell models, it was important to study the contribution of ZEB1 factor to the melanocyte’s differentiation, the spindle cell morphology and their relationship to the invasive type of tumor.

In tumor progression, hypoxia stimulates the expression of the *ZEB1* gene, which increases the motility of tumor cells and stimulates the growth of new blood vessels. Next, the vessel’s growth is regulated by the interaction of vascular endothelial growth factor (VEGF-A) and its receptors (VEGFRs) on endothelial cells. Since ligand sequestering has prevented the activation of VEGFRs, VEGF-targeted anti-angiogenic therapy has been proposed for several cancers. In UM VEGF-A expression is variable. However, the increased concentration of VEGF-A in the aqueous humor, in general, correlates with tumor size and is significantly raised in UM patients with metastases compared with those without metastases [21,22]. Aflibercept (EyleaTM, Bayer), VEGF-A-trapped drug, has being investigated for the treatment of secondary retinopathy in patients after radiotherapy of primary UM [23]. Thus, it is important to study the molecular response of UM to anti-VEGF drugs in a large sample of UM cell lines. Therefore, VEGF-A, in addition to ZEB1, has also become an object of our research.

In this study, it was important to investigate the following questions in UM cell models: (1) Is the level of melanocyte differentiation related to invasiveness? (2) Is ZEB1 expression necessary for the spindle-cell phenotype? (3) How does pharmacological inhibition of VEGF-A affect ZEB1? (4) How does the knockdown of ZEB1 affect UM cell proliferation and VEGF-A production? We believed that the answers to these questions would offer us a way to regulate ZEB1 and VEGF-A in invasive UM.

## 2. Results

### 2.1. UM Tumor Sample Classification

Eight cell cultures of uveal melanoma were prepared from tumor samples, as described in the Methods section. Table 1 represents the characteristics of the initial patient’s tumor cohorts. It can be seen that all cases have a large tumor diameter and this parameter was the indicator for enucleation. Therefore, all cases at the start of the study already had tumor size as an unfavorable metastasis factor. Three melanomas were invasive—uMel3, uMel5 and uMel11.

The parameter of invasive growth of the initial tumor was taken into account for further consideration of cell cultures. Five of the tumors were of spindle-cell morphology and others were of mixed spindle-epithelial morphology. Among them, only one tumor was an invasive type—uMel11. According to its big size, mixed morphology and invasive growth, the uMel11 tumor had the worst metastatic prognosis among the tumor samples.

### 2.2. Cell Culture Morphology and Ultrastructure to Assess the Degree of Differentiation

**Figure 1:**
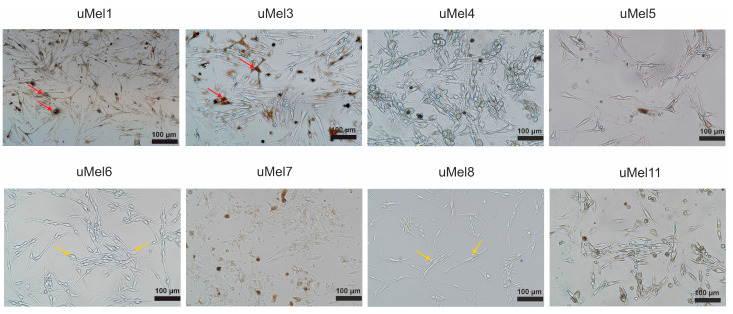
Morphology of obtained UM cells. Phase-contrast images of cells growing as adherent cultures at 2–5 passages. The red arrows indicate examples of well-differentiated melanocytes and the yellow ones indicate low-differentiated ones.

Increased dendricity and pigmentation are the signs of well-differentiated melanocytes. Figure 1 demonstrates the cell culture morphology. uMel1 and uMel3 cells contain pigmented cells with well-developed dendritic extensions and this makes it possible to classify them as well-differentiated melanocytes.

**Figure 2:**
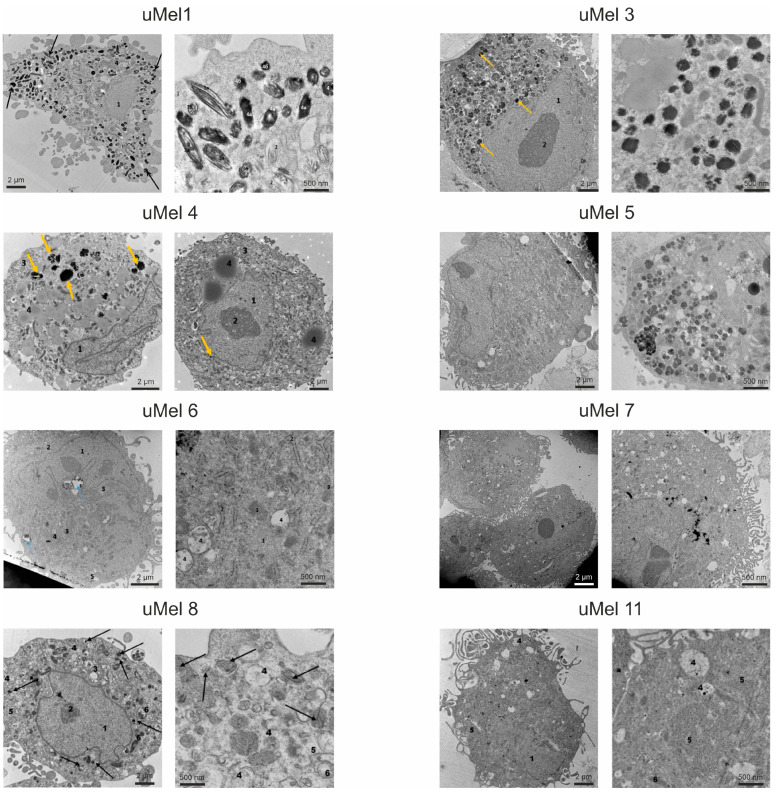
Ultrastructure of UM cells. The characteristic type of melanosomes is additionally represented for uMel1 and uMel3 cells. All arrows indicate melanosomes. Transmission electron microscopy data. To prepare the samples, the cells were detached, fixed and sections of the cell precipitate were made. The following structures are marked with numbers in the images: 1—nucleus, 2—nucleolus, 3—Golgi apparatus, 4—multivesicular bodies/melanosomes, 5—rough endoplasmic reticulum, 6—mitochondria.

Additionally, we analyzed the melanocytes’ differentiation degree based on the mature melanosomes content using transmission electron microscopy (TEM). Full-fledged melanin synthesis requires melanosome maturation into four stages (I-IV) from early endosomes to functional ones [24]. Representative photographs of cells are presented on Figure 2. uMel1 and uMel3 cells contained melanosomes of all stages of maturation—from multivesicular bodies which are morphologically identical to melanosomes of the Ist degree to mature (IVth stage) melanosomes. This allows uMel1 and uMel3 to be classified as the most differentiated. According to melanosome’s structure in all cell lines, only the uMel3 cells produced two types of melanin: pheomelanin and eumelanin. In the remaining cell cultures, there are significantly fewer mature melanosomes, or melanosomes of the “classical” structure were not registered. The least-differentiated cells in terms of the presence of mature melanosomes were uMel11 cells, where there were no visual signs of melanin assembly, accumulation or degradation in all cells.

Thus, additional signs of a negative prognosis of metastasis were revealed only for one of the three lines obtained from invasive tumors: mixed morphology and a low degree of differentiation.

### 2.3. Analysis of ZEB1 Expression in UM Cells

**Figure 3:**
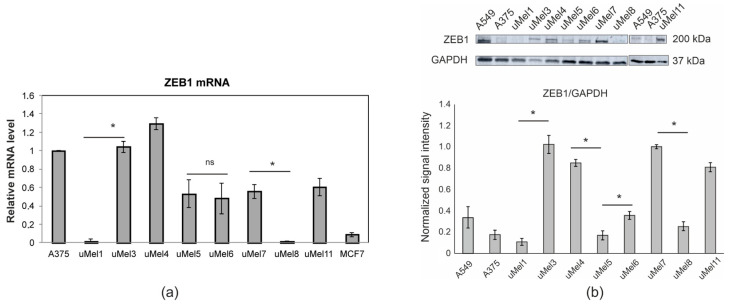
mRNA and protein levels of ZEB1. (**a**) Real-time reverse transcription-PCR analysis of ZEB1 mRNA. The expression of ZEB1 mRNA was normalized to the expression level of beta-2 microglobulin (B2M) mRNA. The expression levels of ZEB1 mRNA in the different cell lines are shown relative to their expression level in A375 cells where it was set equal to one. Data presented as mean values of relative mRNA ± SD of three independent experiments. (**b**) Immunoblot analyses of ZEB1 in UM cells. One representative Western blot of two independent experiments is shown. Quantification of the protein expression was normalized to GAPDH as a loading control. A375 and A549 cell lines were used as relative controls. The protein bands of ZEB1 were quantified as relative values to loading control bands. * The difference between two experimental groups was statistically significant at *p* < 0.05, ns—non-significant (non-parametric Mann–Whitney U test).

ZEB1’s mRNA and protein level were estimated in UM cell lysates (Figure 3a). Six cell lines were ZEB1-positive and two cell lines, uMel1 and uMel8, were ZEB1-negative according to ZEB1 mRNA.

The same PCR product was synthesized in all cell samples (Supplementary Figure S1). ZEB1 mRNA-negative cells were also ZEB1^low^ at the protein level (Figure 3b). uMel5 ZEB1 mRNA-positive cells were ZEB1^low^ at the protein level. We think that such a mismatch between the levels of mRNA and protein may be due to the regulation at the level of translation and/or increased ZEB1 protein degradation in proteasomes in these cells. Thus, three cell cultures had the ZEB1^low^ protein phenotype. All cell cultures with ZEB1^low^ phenotype were obtained from spindle cell tumors—uMel1, uMel5 and uMel8. Thus, the mesenchymal-like spindle-cell phenotype is not associated with high ZEB1 in UM cells. This significantly differentiates UM cells from breast cancer cells [13]. Since not only the cultures obtained from invasive tumors had the ZEB1^high^ phenotype, single ZEB1 does not predict the invasiveness of UM very well.

### 2.4. VEGF Production and the Effect of Its Inhibition in UM Cells

**Figure 4:**
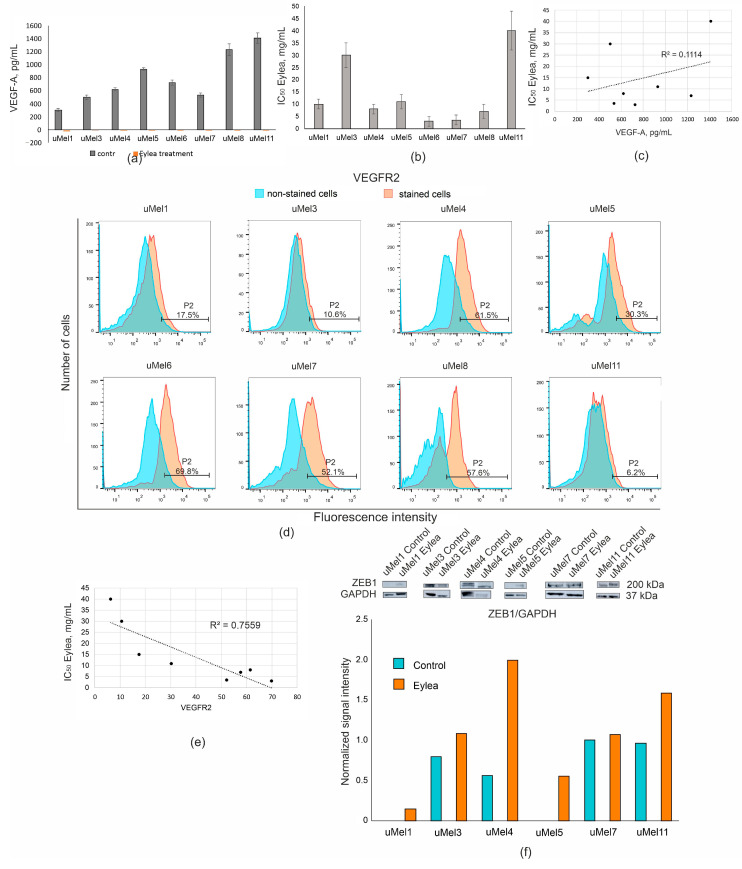
Analysis of VEGF-A concentration, its main receptor VEGFR2 and the sensitivity of UM cells to Eylea. (**a**) ELISA data of VEGF-A concentration in a culture medium of control cells and of cells treated with Eylea (5 mg/mL, 72 h); (**b**) IC_50_ values for UM cells presented as mean values of Eylea concentration ± SD from three independent experiments; (**c**,**e**) graphs represent trend lines with its R^2^ value; and (**d**) evaluation of VEGFR2 receptor (%) in UM cells by flow cytometry. A representative example of the analysis; (**f**) s representative example of ZEB1 analysis in UM cells treated with Eylea (5 mg/mL, 48 h) and its quantification.

We detected VEGF-A in a culture medium of UM cells. UM cells produced VEGF-A at the level of 781 ± 382 pg/mL (Figure 4a). All cells could potentially be susceptible to anti-VEGF-A drugs. Therefore, we determined the cytotoxic activity of anti-VEGF-A drug, Eylea, towards UM cells (Figure 4b). Index cytotoxicity (IC_50_), when 50% of the cell died, revealed that uMel3 and uMel11 cells were resistant to Eylea in the range of 0.2–20 mg/mL, while Eylea, at a concentration of 5 mg/mL, completely bound VEGF-A in the culture medium of all UM cells, according to immunoassay data (Figure 4a). The R-squared Pearson coefficient revealed no correlation between the level of VEGF-A and sensitivity to anti-VEGF-A drug (Figure 4c). Since Eylea does not interact directly with the cell, but rather blocks ligand binding to the receptor, we measured the level of the VEGFR2 receptor, the main VEGF-A receptor on UM cells (Figure 4d). The lowest VEGFR2 was in uMel3 and uMel11 which were the most resistant to cytotoxic effects of Eylea. Cellular VEGFR2 level correlated well with sensitivity to the cytotoxic effects of Eylea (Figure 4e). Thus, Eylea can potentially be considered not only as a drug that prevents neovascularization, but also as a drug with a direct cytotoxic effect for VERFR2^high^ uveal melanomas.

Next, we analyzed how the inhibition of VEGF-A affects ZEB1 levels in UM cells. We found that VEGF-A blocking positively regulated ZEB1 in UM cells. This effect was significant even in ZEB1^low^ cells (Figure 4f). The data obtained indicates that the absence of VEGF-A leads to the stimulation of ZEB1 expression.

### 2.5. Effects of ZEB1 Silencing on UM Cell Proliferation and VEGF-A Synthesis

The aim of the study was also to evaluate whether ZEB1 silencing has a cytotoxic or cytostatic effect on UM cells, taking into account the different ZEB1 mRNA levels. We have demonstrated that 100 nM or 150 nM siRNA efficiently decreased the ZEB1 protein: cell lost up to 55% of ZEB1 24 h after the treatment, and up to 90% by 48 h (Supplementary Figure S2). It is noteworthy that, in our study, increasing the concentration of anti-ZEB1 siRNA to 150 nM did not lead to a further decrease in ZEB1 levels. Therefore, 100 nM siRNA was used for further experiments. The proliferation of siRNA-treated UM cells was evaluated in real time, and random-sequence siRNA (scramble siRNA, siScr) was used as a negative control. It turned out that the effect of siRNA was not entirely predictable based on the ZEB1 level. ZEB1-mRNA-negative cells, uMel1 and uMel8, were resistant to siRNA treatment. The treatment of UM cells with siZEB1 decreased the viability of ZEB1+ mRNA cells: uMel4, uMel5 and uMel6 cells. More interestingly, ZEB1-positive uMel3 and uMel11 cells were also resistant to ZEB1 siRNA, and uMel7 cells proliferation was stimulated by ZEB1 siRNA. Based on the data obtained, it is not clear why cells with high levels of ZEB1 mRNA have a different proliferative response to ZEB1 inhibition. However, these results reduce the potential of ZEB1 as a therapeutic target.

**Figure 5:**
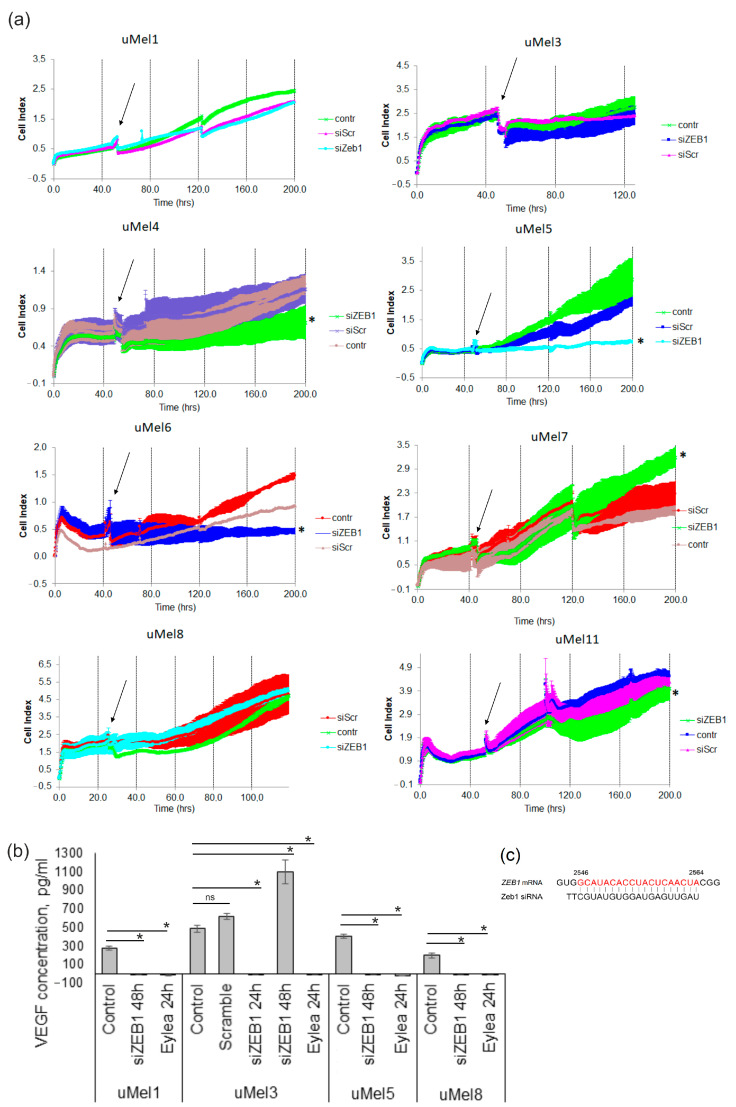
Analysis of ZEB1’s silencing effects on UM cell viability and VEGF-A synthesis. (**a**) Proliferation curve in real time. The differences between the control and experimental groups were significant when * *p*-value < 0.05 (non-parametric Mann–Whitney U test). The arrows indicate when siRNAs were added; (**b**) VEGF-A concentration in the culture medium of UM cells, treated with siZEB1. Eylea treatment was used as a positive control. The differences between the control and experimental groups were significant when * *p*-value < 0.05, ns—non-significant (paired Student’s *t*-test). (**c**) Schematic alignment of Zeb1 siRNA with ZEB1 mRNA. A region of ZEB1 mRNA complementary to 1–19 positions in siRNA is shown in red. Numbers indicate the nucleotide positions in ZEB1 mRNA. The reference sequence was taken from the NCBI database, accession number: NM_001174096.2.

Next, we analyzed whether ZEB1 silencing affects VEGF-A production. We selected uMel1, uMel3 and uMel8 cells in which siZEB1 did not alter viability, and uMel5, where siZEB1 had a cytostatic effect. Cells were treated with siZEB1 for 4 h after that culture medium was replaced for the fresh one. ZEB1 transcription factor is a relatively short-lived protein with a half-life of three hours. Analysis of VEGF-A in culture medium 24 h after siZEB1 treatment showed total suppression of VEGF-A production in UM cells (Figure 5b). Unexpectedly, in cells with the ZEB1^low^ mRNA phenotype, uMel1 and uMel8, VEGF-A production was also inhibited after ZEB1 knockdown. In uMel5 cells, VEGF-A suppression may occur not only as a result of ZEB1 silencing but also as a result of a siZEB1-dependent drop in metabolism under the cytostatic state. The duration of VEGF-A suppression in ZEB1^high^ uMel3 cells was shorter than in ZEB1^low^—complete suppression 24 h after transfection and normalization to the control level with a slight increase after 48 h. The data obtained suggests that the ZEB1 transcription factor is a positive regulator of VEGF-A in UM cells, even in the case of the ZEB1^low^ phenotype.

## 3. Discussion

Long-lived cell cultures obtained from human tissue make it possible to identify new patient-specific cancer markers and to test new drugs. Currently, four UM cell lines from primary tumors are available in the ATCC^TM^ bioresource collection. It poorly reflects the inherent heterogeneity of uveal melanomas. This indicates a task aimed at expanding the number of UM cell lines [25,26]. In this regard, many studies aimed at identifying differences in UMs are carried out using epithelioid-type C918 cells and the spindle-type OCM1 cells [16]. In our work, we used our own panel of eight cell cultures, three of which were obtained from mixed-type tumors and five from spindle-cell tumors. The spindle-shaped phenotype of UM has been known for several decades as a pathological factor in clinics, indicating a decrease in malignancy compared with the aggressive epithelioid phenotype [15]. For various cancers, in addition to changes in the morphology of cells, their poor differentiation is also a sign of an aggressive tumor [27]. For skin melanoma, it is shown that tumors can arise from either melanocyte stem cells or differentiated pigment-producing melanocytes [28]. However, one of the most popular cell models of skin melanoma is of highly aggressive, low-grade, pigment-less melanoma, A375, which reflects only one of the possible phenotypes. Taking this into account, we determined the level of melanocyte differentiation for all cell cultures and showed that the majority, six out of eight cultures, were represented by low-differentiated melanocytes with a low level of dendricity and a small number of mature melanosomes. High-dendricity is inherent in mature melanocytes, as they originate from neural crest cells [29]. Our data indicated that invasive UM, like cutaneous melanomas, can also be presented by highly differentiated pigment-producing melanocytes, as we can see in the case of uMel3 cells. Two other cell cultures, uMel5 and uMel11, obtained from invasive tumors, had extremely low melanocytic differentiation. This indicates that UM malignancy is not always accompanied by loss of differentiation, the opposite to gliomas and carcinomas [9,30,31].

The biological effects caused by ZEB1 expression in UM are still poorly studied. In the work of Chen Y. et al., it was shown on histological samples of patients with mixed-type UM that a ZEB1-positive signal was detected only in the epithelioid cells [15]. Overall, they showed that ZEB1 was highly expressed in UM, and its high expression appeared to be associated with malignancy, but not with the spindle phenotype in UM. This significantly distinguishes UM from breast cancer, where ZEB1-positive tumor cells are of a mesenchymal high-proliferative phenotype. For gliomas, in contrast, new research has shown that ZEB1 loss increases glioma stem cell tumorigenicity and resistance to chemoradiation, and ZEB1 deletion is associated with shorter patient survival [32].

In our study, high levels of ZEB1 mRNA were detected in both spindle and mixed-type cell lines. Cultures with a high ZEB1 mRNA content were obtained from invasive tumors. A comparison of ZEB1 mRNA and protein level showed that, for most cultures, protein levels correlated with mRNA levels. Nevertheless, in uMel5 cells with a high level of ZEB1 mRNA, ZEB1 protein was not detected. We believe that in uMel5 cells, ZEB1 protein synthesis is disrupted or that ZEB1 proteolysis occurred. Such a negative ZEB1 translation regulator has been described for pancreatic ductal adenocarcinoma cells. It was the heterogenic nuclear ribonucleoprotein A1 (hnRNPA1) factor, with nine homologous sequences in the 3′-UTR ZEB1 mRNA [33]. In addition, low activity of the ubiquitin-specific protease 22 (USP22) as a deubiquitinating enzyme may promote ZEB1 degradation in proteasomes [34].

Asnaghi et al. have compared ZEB1 mRNA levels in five uveal melanoma cell lines (OCM1, Mel285, Mel290, OMM1 and 92.1) and in 30 primary tumors. They found that all cell lines have the ZEB1^high^ phenotype, and tumors were represented by samples with ZEB1 levels much higher than in culture, neither were ZEB1-negative [35]. Thus, ZEB1 mismatch between cell cultures and patient tumors was demonstrated. The identification of ZEB1-negative UM cell lines in our study partially closes the existing gap on ZEB1-negative UM cultures. In our study, the comparison of the level of ZEB1, invasiveness, and cellular differentiation of melanocytes showed that ZEB1-positive UM, like uMel3, and ZEB1-negative UM, like uMel1, can both be differentiated, and the level of ZEB1 protein does not predict the invasiveness of UM very well.

**Figure 6:**
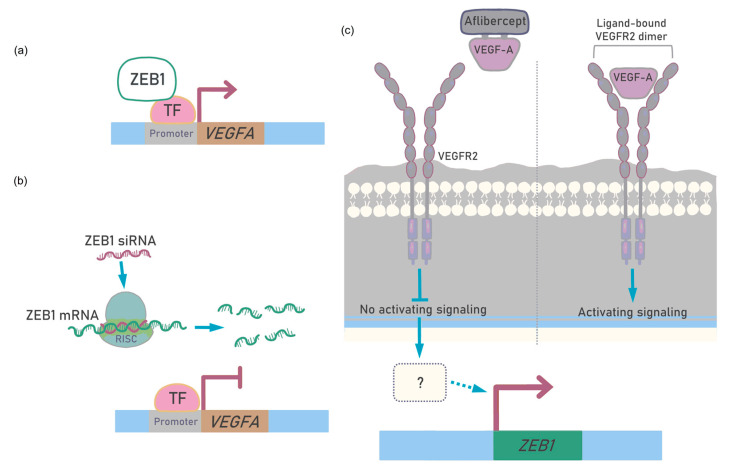
Potential cross-regulation of ZEB1 and VEGF-A. (**a**) ZEB1 activates VEGF-A transcription. (**b**) ZEB1 silencing prevents VEGF-A transcription. (**c**) VEGF-A capturing activates feedback loop resulted in ZEB1 transcription. TF—transcription factor. Figure is made in Sketchbook (2024), version 6.1.1, copyright holder—Sketchbook (https://www.sketchbook.com).

VEGF-A factor is a stimulator of endothelial cell proliferation, and it is this function that is suppressed by VEGF-A inhibitors [36]. It was shown on UM cell cultures that the monoclonal antibody, bevacizumab, targeting all isoforms of VEGF-A, effectively reduced VEGF-A in culture mediums and significantly reduced the proliferation of the 92.1 UM cell line [37]. We have found that VEGF-A inhibitor, Eylea can affect the viability of the UM cells, and sensitivity was inversely correlated with the level of the VEGFR2 receptor. Interestingly, the inhibition of VEGF-A in the culture medium led to the stimulation of ZEB1 expression in UM cells. Since the absence of a ligand prevents signaling by the VEGF-A/VEGFR2 ligand–receptor complex, the compensatory regulatory loop stimulating ZEB1 and ZEB1-dependent VEGF-A expression is most likely activated. It is known that the binding of VEGFA to the ectopic VEGFR2 stimulates the trafficking of intracellular VEGFR2 from the Golgi apparatus to the plasma membrane [38]. Therefore, it is most likely that intracellular VEGFR2 trafficking is involved in the negative regulation of ZEB1. Thus, repression of the negative regulator stimulates ZEB1 expression. It is difficult to make a definitive assumption about the potential cascade that mediates the feedback between VEGF-A depletion and ZEB1 stimulation. Such regulation is also possible through intermediators, for example, those that do not receive a signal from the kinase activity of VEGFR2 after activation by the ligand. An alternative may be negative regulation of microRNAs from mir-200 family resulting from a decrease in VEGF-A concentration [39]. We attempted to schematically represent the effects we observed in Figure 6.

Based on the data received, the possible transition of UM cells after anti-VEGF-A treatment from ZEB1^low^ to the ZEB1^high^ state can be considered a negative factor for the use of Eylea for the UM therapy. However, for some types of cancer, such as head and neck carcinoma, it has been shown that ZEB1 overexpression increased the tumor susceptibility to ferroptosis and anti-VEGF-A treatment can be considered as an option for use in combination with ferroptosis inducers [40].

We also showed that ZEB1 inhibition negatively regulates VEGF-A in UM cultures, both in ZEB1^high^ and ZEB1^low^ cells. These data are consistent with the data obtained by Liu et al. for breast cancer, where ZEB1 caused a marked upregulation of the expression of VEGF-A at both mRNA and protein levels [41]. Mechanistically, this may be a complex regulation, and for skin wound healing, ZEB1 has been shown to upregulate VEGF-A via microRNA-206 suppression [42]. In hepatocellular carcinoma, ubiquitin-specific protease 22 (USP22) has been shown to stabilize ZEB1 and thereby upregulate ZEB1-mediated VEGF-A transcription [34]. However, it is worth considering that ZEB1 is not the only regulator of VEGF transcription, which complicates the interpretation of the data.

Since many researchers consider high ZEB1 as a negative factor for both skin melanoma and UM, various attempts have been made to regulate ZEB1 and validate it as a therapeutic target [15,43]. We tested the effect of ZEB1 inactivation on UM cell proliferation using siZEB1 silencing. Most cell lines with high ZEB1 mRNA had reduced proliferation in response to silencing while ZEB1-low cells were resistant to siZEB1. However, there were cells with a ZEB1^high^ phenotype that increased proliferation in response to ZEB1 inhibition, as we observed for uMel7 cells.

## 4. Materials and Methods

### 4.1. Chemicals and Antibodies

Alpha-MEM medium (α-MEM), Opti-MEM™ reduced serum medium, fetal bovine serum FBS, L-glutamine, TrypleTM and 10× antibiotic/antimicotic (a/a) containing 1000 u/mL of penicillin, 1000 mg/mL of streptomycin and 2 µg/mL of amphotericin, were all from GIBCO, Thermo Fisher Scientific, Waltham, MA, USA. Insulin and Epidermal Growth Factor (EGF) were from SajStorLab, Moscow, Russia. Anti-human ZEB1 antibodies (#A25437) and anti-human GAPDH antibodies (#AC033) were from ABclonal, Wuhan, China. Conjugates of antibodies with horseradish peroxidase against rabbit IgG (#G-21234) and mouse IgG (#G-21040), PE Mouse Anti-Human CD309 (VEGFR-2) (#89106) were from BD Pharmingen (San Diego, CA, USA) and the substrate, Novex ECL HRP was from Invitrogen, Carlsbad, CA, USA. Eylea (Aflibercept) was from Bayer/Regeneron Pharmaceuticals, Leverkusen, Germany.

### 4.2. Tumors

All 8 patients who underwent enucleation histopathology confirmed the clinical diagnosis of UM and cell type (spindle or mixed). The cohort included tumors that were not suitable in size for organ-preserving conservation therapy, including brachytherapy (prominence h > 9 mm). Preliminary UM diagnosis, tumor prominence and tumor topographic characteristics were evaluated using ocular ultrasonography (B-scans). No distant metastases were detected in any of the patients at the time of enucleation. The mean age of all subjects was 52 years (range 41–68) and 75% were male. The study was conducted according to the guidelines of the Declaration of Helsinki. Study design was approved by The Ethics Committee of S. Fyodorov Eye Microsurgery Federal State Institution Novosibirsk branch (reference 2023 #1, 14 June 2023). Tumor samples were obtained with informed consent from patients at the S. Fyodorov Eye Microsurgery Federal State Institution, Novosibirsk branch (Novosibirsk, Russian Federation).

### 4.3. Cell Culture Preparation

Personal UM cell cultures were obtained as previously described in [19,44] with minor modifications. In short, a small piece of fresh tumor, approximately 5 mm^3^, was finely minced with a sterile scalpel blade in PBS. The minced fragments were placed in the wells of a 24-well plate in an α-MEM with nucleosides with the following additives: 10% FBS, 2 mM glutamine, 100 u/mL of penicillin, 100 mg/mL of streptomycin, 0.25 µ/mL of amphotericin, 5 µg/mL of insulin and 20 ng/mL of EGF and stayed at 37 °C in a humidified atmosphere containing 5% CO_2_. Every 48 h, the tissue fragment from which the cells migrated to the surface of the well were transferred to a new well with fresh culture medium. This procedure was repeated up to 5 times, and after that, the cells were grown to a monolayer, detached with Tryple, combined and cultivated in 25 cm^2^ flask (TTP, Basel, Switzerland). On the 2nd to 3rd passages, a cell’s biobank was formed, where cells were stored at −70 °C in a liquid nitrogen atmosphere.

### 4.4. Transmission Electron Microscopy (TEM)

All reagents for TEM were purchased from EMS (Hatfield, PA, USA). Cells were collected by centrifugation at 6000 rpm for 5 min, fixed with 4% paraformaldehyde for 24 h and postfixed with 1% osmium tetroxide for 1 h. The samples were routinely dehydrated in ethanol and acetone and embedded into an epon–araldite mixture to obtain hard blocks. Ultrathin sections were prepared using a diamond knife (Diatome, Nidau, Switzerland) on an ultramicrotome EM UC7 (Leica, Wetzlar, Germany), routinely contrasted with uranyl acetate and lead citrate and examined using a JEM 1400 transmission electron microscope (JEOL, Tokyo, Japan). Images were acquired with a Veleta digital camera (EM SIS, Münster, Germany).

### 4.5. RNA Extraction and Real-Time Reverse-Transcription Polymerase Chain Reaction (RT-PCR)

Cells (1 × 10^6^) were lysed with the Lira reagent (Biolabmix, Novosibirsk, Russia) for 10 min at room temperature. RT-PCR was performed with the BioMaster RT-PCR SYBR Blue mix (Biolabmix, Russia) using the LightCycler 96 System (Roche, Mannheim, Switzerland), as was described in [45]. Contaminating genomic DNA was removed from the samples by DNase I (BioLabMix, Russia) treatment. A weight of 40 ng of DNA-free RNA was used for the reaction. mRNA relative levels were calculated using LightCycler 96 software and relative values were normalized to the levels of beta-2 microglobulin (B2M) reference gene mRNA. The gene-specific primers were as follows, in Table 2.

### 4.6. siRNAs

Ribo-oligonucleotides were synthesized in the laboratory of RNA chemistry of the ICBFM SB RAS, Novosibirsk, Russia. We used the following siRNA: siZEB1 SenZeb1 5′-GCAUACACCUACUCAACUATT-3′ and AntiZeb1 5′-UAGUUGAGUAGGUGUAUGCTT-3′ and siScr senScr 5′-CAAGUCUCGUAUGUAGUGGUU-3′ and antiScr 5′-CCACUAUAUACG AGACUUGUU-3′, described in [46]. siRNA hybridization was made as described in [46]. For transfection of siRNAs into UM cells, Lipofectamine 3000 (Invitrogen, USA) was used in accordance with the manufacturer’s recommendations. In order to decrease the expression of ZEB1, we transfected ZEB1 siRNA with the final concentration at 100 nmol in an OPTI-MEM medium and incubated cells for 4 h at 37 °C. Then, the medium was replaced with a complete α-MEM and the cultivation was continued.

### 4.7. Flow Cytometry

For VEGFR2 detection, the cells (1 × 10^6^) were incubated with PE-conjugated anti- VEGFR2 antibodies (1:300) for 1 h at 23 °C. Antibody-stained cells were initially gated based on forward scatter (FS) versus side scatter (SS) to exclude small debris. All the tests were performed using FACSCanto II flow cytometer (BD Biosciences, Franklin Lakes, NJ, USA), and the data were analyzed by BD FACSDiva^TM^ Software v. 6.1.3 (BD Biosciences, Franklin Lakes, NJ, USA). At least 10,000 events were collected in each experiment.

### 4.8. Western Blot

Cells (1 × 10^6^) were lysed with cell lysis buffer (50 mM Tris, pH 8.0, 5 mM EDTA and 150 mM NaCl) containing 0.1% SDS, 1× complete protease inhibitor cocktail (Roche Diagnostics GmbH, Mannheim, Germany) and 1 mM PMSF (Sigma-Aldrich, St. Louis, MO, USA). Western blot analysis of cell lysates was performed as described previously [47]. Signal visualization was performed using conjugates of antibodies with horseradish peroxidase and Novex ECL HRP substrate.

### 4.9. Cell Viability Assays

Cell viability was detected 72 h after the treatment using the MTT test or in real-time mode with the iCELLigence system (ACEA Biosciences Inc., San Diego, CA, USA), as was described previously [48]. IC_50_ values were calculated with CompuSyn version 1.0.1 (ComboSyn, Inc., Paramus, NJ, USA).

### 4.10. ELISA

For the quantitative determination of VEGF-A in the culture medium, we used enzyme-linked immunoassays (#A-8784) from VECTOR Best, Novosibirsk, Russia. Cells (5 × 10^4^) were seeded in the wells of 24-well plates. The samples were prepared according to the manufacturer’s protocols and their optical density was detected with a multichannel spectrophotometer, Apollo LB912 (Berthold Technologies, Oak Ridge, TN, USA) at 450 nm (reference wavelength 620 nm).

### 4.11. Statistics

Significance between two groups was determined using a two-tailed Student’s *t*-test or using a non-parametric Mann–Whitney U test for small sample size with the inability to reliably assess normality and homogeneity of variance in a limited number of replicates. All the error bars represent the standard error of the mean. Pearson’s correlation coefficient was analyzed to reveal the correlation between two data sets.

## 5. Conclusions

UMs with an invasive growth type can maintain a high degree of melanocyte differentiation and various ZEB1 levels. Anti-VEGF drugs can potentially stimulate ZEB1 expression in UM cells and, as a result, ZEB1-dependent invasion. The data obtained allows us to consider ZEB1 suppression as an auxiliary link for a combined strategy of killing UM cells. To confirm the hypothesis, appropriate studies on animal UM models should be undertaken in the near future.

## Figures and Tables

**Table 1:** Characteristics of the tumor at the time of enucleation.

Tumor (Cells)	Invasiveness	Tumor Type	LBD *, mm	Level of Differentiation
uMel1	-	spindle B	15.9	high
uMel3	sprouting into the sclera	spindle B	20.9	high
uMel4	-	mixed	18.9	med
uMel5	sprouting of the ciliary body, eye membranes, the orbital fiber and the optic nerve	spindle	21.0	low
uMel6	-	mixed	9.1	low
uMel7	-	spindle	15.8	low
uMel8	-	spindle	16.8	high
uMel11	sprouting into the sclera	mixed	19.7	non-differentiated

* According to ultrasonography data; LBD—largest basal diameter; Mixed: spindle-epithelioid type.

**Table 2:** Oligonucleotide sequences and alignment.

Gene	Primer Sequence	Reference Sequence Positions Matching Primer Sequence	Exons of a Reference Sequence	NCBI Reference Sequence
*B2M*	For: 5′-TGGGTTTCATCCATCCGACA-3′	174–193	2	NM_004048.4
	Rev: 5′-CGGCATCTTCAAACCTCCAT-3′	418–399	3/4 *	
*ZEB1*	For: 5′-GCAGTCCAAGAACCACCCTT-3′	2576–2595	7	NM_001174096.2
	Rev: 5′-CATGAGGTCTTTTACCTGTGTGT-3′	2819–2797	8/9	

* Exon–exon junction. For: forward, Rev: reverse.

## Data Availability

All data used to support the findings of this study is available from the corresponding author upon request.

## Supplementary Materials

The following supporting information can be downloaded at: https://www.mdpi.com/article/10.3390/ijms262110346/s1.

## Abbreviations

α-MEM	Alpha-MEM medium
ATCC	American type culture collection
B2M	Beta-2-microglobulin
ECL	Enhanced chemiluminescence
EGF	Epidermal growth factor
ELISA	Enzyme-linked immunosorbent assay
EMT	Epithelial-to-mesenchymal transition
FBS	Fetal bovine serum
GAPDH	Glyceraldehyde-3-phosphate dehydrogenase
hnRNPA1	Heterogenic nuclear ribonucleoprotein A1
HRP	Horseradish peroxidase
LBD	Large basal diameter
MTT	3-(4,5-dimethylthiazol-2-yl)-2,5-diphenyltetrazolium bromide
PBS	Phosphate-buffered saline
PE	Phycoerythrin
RT-PCR	Quantitative real-time PCR
SDS	Sodium dodecyl sulfate
siRNA	Small interfering RNA
TEM	Transmission electron microscopy
TF	Transcription factor
UM	Uveal melanoma
USP22	Ubiquitin-specific protease 22
VEGF-A	Vascular endothelial growth factor A
VEGFR2	Vascular endothelial growth factor receptor 2
ZEB1	Zinc finger E-box binding homeobox 1
