# Supplementary material for: ZEB1 and Uveal Melanoma Invasiveness
Source: Int J Mol Sci. 2025 Oct 24;26(21):10346. doi: 10.3390/ijms262110346 (PMC12607405; doi:10.3390/ijms262110346)
Supplement: Supplementary file 1 [file ijms-26-10346-s001.zip › ijms-3911613-supplementary.pdf]

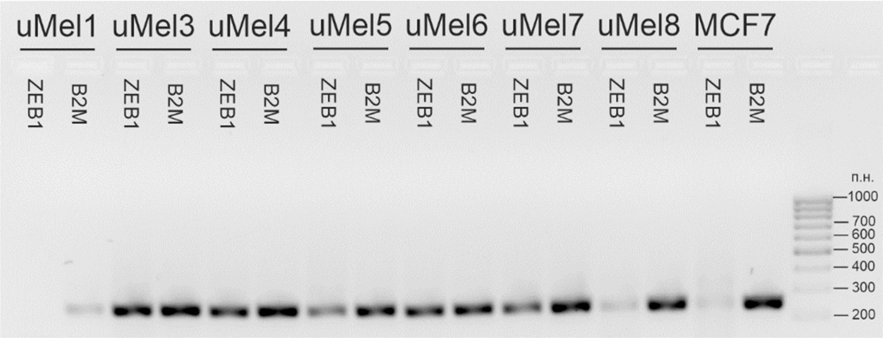

Figure S1. Analysis of ZEB1 mRNA PCR product in agarose gel.

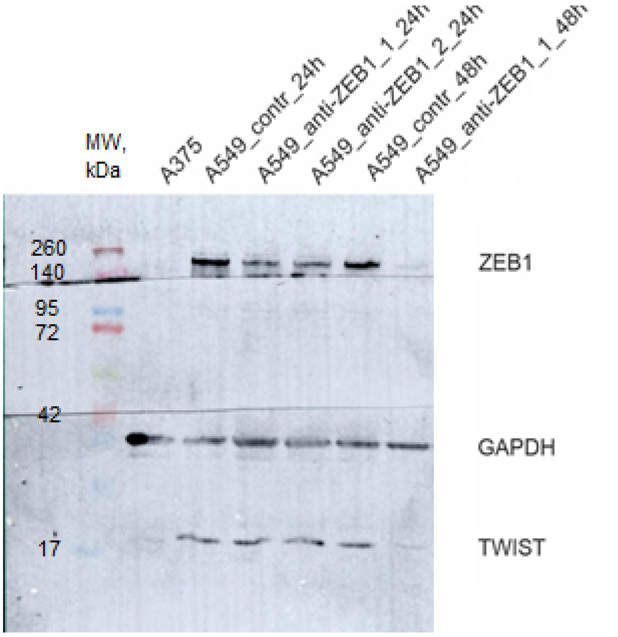

Figure S2. Analysis of ZEB1 silencing in ZEB1-positive A549 lung adenocarcinoma cells 24 h and 48 h after the treatment. A375 were used as negative control. Two concentration of siRNA were used for knockdown: 100 nM (anti-ZEB1-1) and 150 nM (anti-ZEB1 -2).
